# Impairment in S-phase entry of splenocytes of *Parp-1* knockout mice

**Published:** 2004-06-01

**Authors:** Fumiaki Watanabe, Mitsuko Masutani, Nobuo Kamada, Hiroshi Suzuki, Hitoshi Nakagama, Takashi Sugimura, Hirobumi Teraoka

**Affiliations:** *) Department of Pathological Biochemistry, Medical Research Institute, Tokyo Medical and Dental University, 2-3-10 Kandasurugadai, Chiyoda-ku, Tokyo 101-0062, Japan; **) Biochemistry Division, National Cancer Center Research Institute, 1-1 Tsukiji 5-chome, Chuo-ku, Tokyo 104-0045, Japan; ***) Chugai Research Institute for Medical Science, Inc., 1-135, Komakado, Gotemba, Shizuoka 412-0038, Japan; ****) National Research Center for Protozoan Diseases, Obihiro University of Agriculture and Veterinary Medicine, Obihiro, Hokkaido 080-8555, Japan

**Keywords:** Poly (ADP-ribose) polymerase, cell-cycle, splenocytes, S-phase, 3-aminobenzamide, BM Condimed ^R^H1

## Abstract

One immediate cellular response to DNA damage is the polyADP-ribosylation reaction by poly(ADP-ribose) polymerase-1 (Parp-1). The importance of Parp-1 has been established in many cellular processes, such as the maintenance of genomic stability, DNA repair and cell-death induction. Here, we established *Parp-1*^−/−^ mice of C57BL/6J congenic strain and characterized the role of Parp-1 in cell-cycle progression. In this study, we also improved a method to observe G0/G1 to S-phase transition of splenocytes and bone marrow cells prepared from mice. The cells were cultured and stimulated with mitogens (50 μM ionomycin/1 μM phorbol 12, 13-dibutyrate). We found that addition of a commercially available growth supportive reagent, BM Condimed ^R^H1, greatly enhanced the transition of G0/G1 to the S-phase, which was determined by bromodeoxyuridine (BrdU) incorporation to DNA. Using this method, G0/G1 to the S-phase entry was measured using splenocytes derived from *Parp-1*^−/−^, *Parp-1*^+/−^ and wild-type (*Parp-1*^+/+^) mice. DNA synthesis in *Parp-1*^+/+^ and *Parp-1*^+/−^ splenocytes started from day 1 after addition of mitogens, whereas that in *Parp-1*^−/−^ cells started from day 2. The peak of the S-phase was at day 2 in all genotypes and notably DNA synthesis in *Parp-1*^−/−^ cells was approximately halved compared to *Parp-1*^+/+^ cells on day 2, 3 and 4. These results suggested that Parp-1 is involved in positive regulation of S-phase entry in quiescent mouse splenocytes.

## Introduction

Protein polyADP-ribosylation is one post-translational modification on various nuclear and centrosomal proteins.1),2) The synthesis of poly(ADP-ribose) from *β*-NAD^+^ in response to DNA strand breaks is mostly catalyzed by Parp-1,1),2) which is localized in nuclei and centrosomes.3)–5) *In vitro* and *in vivo* experimental studies, including gene-disruption studies, indicate Parp-1 as an active participant in regulation of genomic stability,6),7) DNA repair,8) gene expression9),10) as well as in cellular differentiation11)–13) and cell-death induction14). Furthermore, involvement of Parp-1 on DNA replication and cell cycle regulation, including G0/G1 to S-phase transition,15) G1 and G2 arrest after DNA damages16),17) were also suggested.

On the other hand, another DNA strand break-sensing molecule, the DNA-PKcs-Ku70/80 complex (DNA-PK complex), is also involved in the regulation of cell-cycle progression18) as well as in DNA strand break repair. Recently, we reported the involvement of DNA-PK complex in the control of cell-cycle progression at G1 to S-phase transition by affecting the activity of a transcription factor, E2F.18) Therefore, both Parp-1 and DNA-PK complex, which directly recognize DNA damage, are involved in the cell-cycle checkpoint control. Furthermore, the coordinative interaction between Parp-1 and DNA-PK complex is reported.19),20) Parp-1 stimulates DNA-PK activity19) whereas DNA-PK inhibits Parp-1 activity.20)

To understand the function of the Parp-1 as well as DNA-PK complex on cell-cycle progression, various experimental systems need to be used because the function of these proteins might be different among tissues and cell types. In the present study, we investigated the S-phase entry of quiescent splenocytes of *Parp-1*^−/−^ mice. We unexpectedly succeeded in a marked improvement of the stimulation of S-phase entry in splenocytes and bone marrow cells using BM Condimed ^R^H1. Using this modified method, we demonstrated that *Parp-1* deficiency negatively affects G0/G1 to S-phase transition.

## Materials and methods

### Preparation and culture conditions of splenocytes, bone marrow cells and thymocytes

**Fig. 1:**
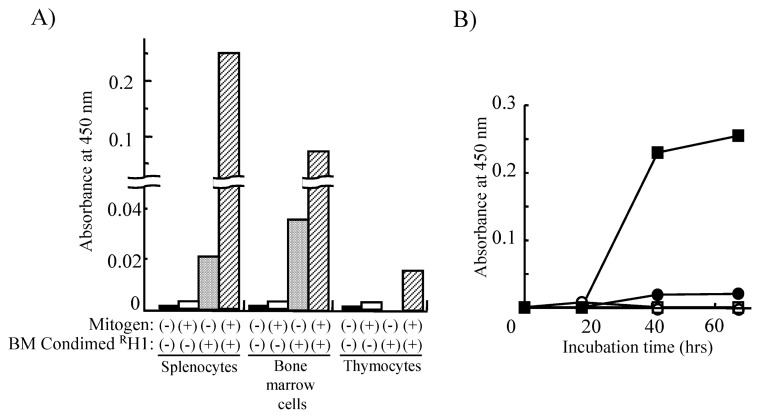
DNA synthesis in mitogen-stimulated splenocytes, bone marrow cells and thymocytes prepared from wild-type mice of 5-week-old. (A) The effect of BM Condimed ^R^H1 and mitogen stimulation on splenocytes, bone marrow cells and thymocytes from wild-type mice. The cells were cultured in the presence (gray and hatched bars) or absence (closed and open bars) of BM Condimed ^R^H1. DNA synthesis in these cells was analyzed 2 days after mitogen stimulation (open and hatched bars) or without stimulation (closed and gray bars). All the experiments were repeated twice. (B) Time course of DNA synthesis in the presence or the absence of BM Condimed ^R^H1. Splenocytes from wild-type mice were cultured in the presence (closed and open squares) or absence (closed and open circles) of BM Condimed ^R^H1. BrdU incorporation into DNA was analyzed at the indicated time points after mitogen-stimulation (closed squares and closed circles) or without stimulation (open squares and open circles).

**Fig. 2:**
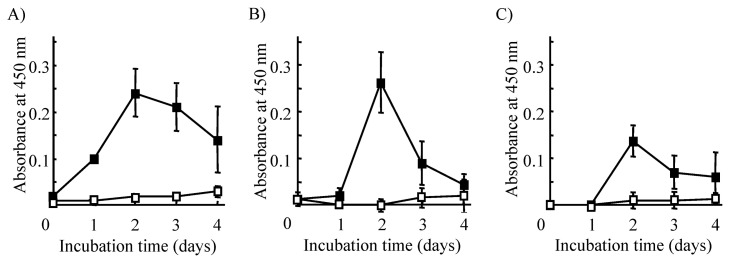
DNA synthesis in mitogen-stimulated splenocytes prepared from *Parp-1*^+/+^, *Parp-1*^+/−^ and *Parp-1*^−/−^ mice. BrdU incorporation into DNA in splenocytes from *Parp-1*^+/+^ (A), *Parp-1*^+/−^ (B) and *Parp-1*^−/−^ (C) mice were analyzed at the indicated time points after mitogen-stimulation (closed squares) or without stimulation (open squares). Splenocytes were prepared from mice at the age of 12-week-old. Data are presented as means ± S.E. of three mice.

All animals were maintained in a room controlled for 12 hrs light/dark cycle, temperature (25°C), and humidity (50±10%). *Parp-1*^−/−^ mice harbor exon 1 disruption through the insertion of a neomycin resistance gene cassette.21) *Parp-1*^−/−^ congenic mice of C57BL/6J genetic background were established by backcrossing the mice of ICR/129Sv mixed genetic background for 8 successive generations with C57BL/6J mice. Splenocytes, bone marrow cells and thymocytes were isolated from 5-week-old male C57BL/6J mice as previously described22) and plated at a density of 1×10^5^ cells in 0.1-ml culture into 96-well plates for the experiment in Fig. 1. Splenocytes of *Parp-1*^−/−^, *Parp-1*^+/−^ and wild-type (*Parp-1*^+/+^) male mice were isolated at 12-week of age and plated as above for the experiment in Fig. 2. These cells were cultured in RPMI 1640 supplemented with 10 % fetal bovine serum and BM Condimed ^R^H1 (Boehringer Mannheim) and were stimulated with 0.5 μM ionomycin/10 nM phorbol 12, 13-dibutyrate for 1, 2, 3 and 4 days22) in a triplicate manner. It is described that BM Condimed ^R^H1 is prepared from the supernatant of a mouse thymoma cell line stimulated with 12-*O*-tetradecanoylphorbol 13-acetate and contains a complex mixture of growth factors and cytokines.23) It contains 15% fetal calf serum, 1 mM oxalacetate, 1 mM sodium pyruvate, 0.2 mg/ml insulin, 1 ng/ml human interleukin 6 and 10 ng/ml 12-*O*-tetradecanoylphorbol 13-acetate.

### Measurement of DNA synthesis in splenocytes, bone marrow cells and thymocytes

Bromodeoxyuridine (BrdU) was added into culture medium 2 hrs before harvesting cells. BrdU incorporation into DNA was measured subsequently by enzyme immunoassay using an ELISA kit (Boehringer Mannheim) as previously described.24)

## Results and discussion

We established *Parp-1*^−/−^ congenic mice of the C57BL/6J strain. Brother-sister matings of *Parp-1*^+/−^ mice produced offspring at the ratio of 4 (*Parp-1*^+/+^):5 (*Parp-1*^+/−^):1(*Parp-1*^−/−^) (total number of offspring was 60). Thus a decreased ratio of *Parp-1*^−/−^ offspring than Mendelian ratio was observed, although *Parp-1*^−/−^ mice were fertile. When E13.5 embryos obtained from brother-sister mating of *Parp-1*^+/−^ mice were genotyped, *Parp-1*^−/−^ embryos (6/21) were observed in a Mendelian fashion, suggesting that *Parp-1* deficiency could possibly affect the embryogenesis later than this stage.

To investigate *de novo* DNA synthesis in mitogenstimulated cells using ELISA, we isolated splenocytes, bone marrow cells and thymocytes from wild-type mice. When these cells were exposed to the mitogens, 50 μM ionomycin/1 μM phorbol 12, 13-dibutyrate, in the absence of BM Condimed ^R^H1, the DNA synthesis measured by BrdU incorporation was hardly detectable. However, addition of BM Condimed ^R^H1 increased DNA synthesis 85-fold in splenocytes and 31-fold in bone marrow cells. On the other hand, DNA synthesis of thymocytes was only augmented 8-fold (Fig. 1A and 1B). Because BM Condimed ^R^H1 contains specific growth factors for B cells,23) it is possible that the B-cell population in the splenocytes and bone marrow cells was influenced but the T-cell population, which is the main component of thymocytes, was not affected. These results indicated that BM Condimed ^R^H1 is useful for detection of *de novo* DNA synthesis in mitogen-stimulated splenocytes and bone marrow cells.

Using this novel modified method, we measured DNA synthesis of splenocytes from *Parp-1*^+/+^, *Parp-1*^+/−^ and *Parp-1*^−/−^ mice after mitogen stimulation, as shown in Fig. 2. DNA synthesis in *Parp-1*^+/+^ and *Parp-1*^+/−^ splenocytes was detected from day 1 after mitogen stimulation, whereas that in *Parp-1*^−/−^ splenocytes started at day 2 after mitogen stimulation. DNA synthesis peaked at day 2 in all genotypes, however, DNA synthesis in *Parp-1*^−/−^ splenocytes was almost halved compared to that in *Parp-1*^+/+^ cases on day 2, 3 and 4. It is notable that *Parp-1*^+/−^ splenocytes also showed a lowered level of DNA synthesis at day 3 and 4, as in *Parp-1*^−/−^ splenocytes. These results led to the conclusion that Parp-1 is involved in the regulation of the cell cycle when quiescent splenocytes at G0/G1-phase enter the S-phase. Since the length of the transition time from G0/G1 to the S-phase was not influenced in *Parp-1*^−/−^ animals compared to *Parp-1*^+/+^ and *Parp-1*^+/−^ animals, it is suggested that *Parp-1* deficiency lowered the frequency of S-phase entry in splenocytes after mitogen-stimulation, but did not alter the sequential cascade for S-phase entry itself. It is also possible that process of DNA synthesis in S-phase in *Parp-1*^−/−^ splenocytes may be slower than in *Parp-1*^+/+^ cells.

Our findings are consistent with the result reported by Rosenthal *et al.* on mouse embryonic fibroblasts.15) They reported that G0/G1 to S-phase entry in serum-starved cells is decreased and that the expression of necessary genes in G1 to S-phase transition depending on E2F-1 promoter activity is lowered in *Parp-1*^−/−^ embryonic fibroblasts.

In addition, we also investigated the effect of a polyADP-ribosylation inhibitor 3-aminobenzamide (3-AB) on *de novo* DNA synthesis in wild-type (*Parp-1*^+/+^) splenocytes. DNA synthesis in the splenocytes after mitogen stimulation in the presence or the absence of 4 mM 3AB was 0.20± 0.045 and 0.15± 0.036 Abs_450_, respectively, and no significant difference was observed. Thus, inhibition of Parp-1 activity did not result in the impairment of DNA synthesis. This suggests the presence of Parp-1 protein itself, but not polyADP-ribosylation, is necessary for G0/G1 to S-phase entry of splenocytes. Since Parp-1 is known to interact with various proteins involved in cell-cycle progression through the BRCT (BRCA-1 C-terminus) domain,25) the protein-protein interaction may be important for the stimulating effect of Parp-1 on G0/G1 to S-phase transition.

The improved method to detect S-phase entry of splenocytes and bone marrow cells should be useful for further characterization of the role of Parp-1 as well as other regulatory molecules in the process of G0/G1 to S-phase transition.
